# Enhanced Visual Attentional Modulation in Patients with Inherited Peripheral Retinal Degeneration in the Absence of Cortical Degeneration

**DOI:** 10.1155/2019/8136354

**Published:** 2019-06-25

**Authors:** Sónia Ferreira, Andreia Carvalho Pereira, Bruno Quendera, Aldina Reis, Eduardo Duarte Silva, Miguel Castelo-Branco

**Affiliations:** ^1^Coimbra Institute for Biomedical Imaging and Translational Research, CIBIT, ICNAS-P, CNC.IBILI, Faculty of Medicine, University of Coimbra, 3000-548 Coimbra, Portugal; ^2^Sackler Institute for Translational Neurodevelopment, Department of Forensic and Neurodevelopmental Science, Institute of Psychiatry, Psychology, and Neuroscience, King's College London, UK; ^3^CNC.IBILI, Institute of Nuclear Sciences Applied to Health, ICNAS, University of Coimbra, 3000-548 Coimbra, Portugal; ^4^Ophthalmology Unit, Centro Hospitalar e Universitário de Coimbra, 3000-075 Coimbra, Portugal

## Abstract

The role of attentional mechanisms in peripheral vision loss remains an outstanding question. Our study was aimed at determining the effect of genetically determined peripheral retinal dystrophy caused by Retinitis Pigmentosa (RP) on visual cortical function and tested the recruitment of attentional mechanisms using functional magnetic resonance imaging (fMRI). We included thirteen patients and twenty-two age- and gender-matched controls. We analyzed cortical responses under attentional demands and passive viewing conditions while presenting a visual stimulus covering the central and paracentral visual field. Brain activity was studied in visual areas V1, V2, and V3 as well as in cortical regions of interest corresponding to the preserved and the damaged visual field. The influence of visual field extent and age of disease onset were also investigated. Cortical thickness of visual areas was also measured. We found that cortical visual responses under attentional demands were increased in patients with larger degeneration of visual field, as demonstrated by significant interaction effects between group and task conditions. Moreover, activation during the task condition was increased for patients in two cortical regions of interest corresponding to the preserved and damaged visual field, specifically in patients with severe visual field loss. These findings were observed in the presence of preserved visual cortical structure. We conclude that RP patients have enhanced visual attention recruitment despite their retinal degeneration, while cortical structure and overall response levels remain intact. The unmasking of feedback signals from higher level visual regions involved in attentional processes may explain the increased cortical responses. These findings are relevant for the design of strategies for treating retinal diseases, based on attentional cuing.

## 1. Introduction

Human studies concerning the effects of peripheral retinal loss on adult visual cortical structure and function are scarce. Previous studies have mainly addressed central retinal disorders such as macular degeneration or other hereditary retinal dystrophies and diseases such as glaucoma [1–3]. Peripheral and central visual information is differentially routed in the brain [4, 5]. Thus, neural adaptation mechanisms might differ when central or peripheral visual degeneration occurs. Previously, we found evidence for visual retinotopic reorganization in RP (peripheral regions responding to more central representations) [6]. It is nevertheless also important to assess the impact of peripheral retinal loss on visual attentional mechanisms. This may provide useful information in the context of low and high level strategies for treating different retinal diseases. Additionally, there is still an ongoing debate on the nature of adult brain functional reorganization induced by retinal diseases [7–10].

Our study is aimed at determining the interaction between attentional mechanisms and peripheral retinal dystrophy caused by Retinitis Pigmentosa (RP) on brain function using magnetic resonance imaging (MRI). RP is an inherited degeneration of photoreceptors that initially affects the peripheral retina and later advancing towards the central retina. The onset age varies from infancy to adulthood. The disease manifestations comprise night blindness, tunnel vision, and possibly blindness in severe stages [11, 12]. In a functional magnetic resonance imaging (fMRI) case series study of three RP patients [13], authors reported task-dependent changes in cortical responses in the lesion projection zone (LPZ—the cortical region that no longer receives input due to a bilateral retinal lesion or scotomata [1]). They suggested the unmasking of feedback signals from higher-order visual areas under attentional demands when retinal input signals are lost. Another fMRI report with one RP patient found no evidence of functional alterations in the LPZ [14]. Our recent study in a relatively large cohort showed clear topological evidence for reorganization, which was dependent on the long-term extent of visual loss [6].

Contrasting with RP, central vision is primarily affected in macular degeneration. Some authors claimed that the deafferented cortical neurons in the primary visual cortex become responsive to inputs from the peripheral retina in this pathology [15–20]. However, other studies have questioned such visual cortical alterations by reporting the existence of a silent LPZ [14, 21–25]. Some of the previous studies showed that visual cortical alterations in macular degeneration are associated with the severity of retinal function loss, arguing that large-scale reorganization only occurs when there is a complete foveal visual loss [15, 18, 20]. However, other researchers did not find any signs for cortical reorganization in a large cohort of patients without foveal sparing [22]. Moreover, the influence of age of disease onset on the degree of cortical alterations is not clear [15, 22], although there is some evidence for larger reorganization in macular degeneration patients with earlier forms of the disease [19]. The reduced numbers of participants [21, 25] and the difference in stimuli and tasks used [26] may have also contributed to the controversy in the reported macular degeneration studies ([1], [7].

Our study was aimed at investigating the effect of peripheral retinal dystrophy caused by RP on brain attentional mechanisms using fMRI taking into account the effect of visual field extent and age of disease onset. Our hypothesis stated that, in addition to the previously demonstrated reorganization, visual cortical responses were also altered as a function of attentional demands in RP patients due to the lack of peripheral retinal bottom-up input. Moreover, we hypothesized that these alterations were more prominent for RP patients with more constricted visual fields and earlier disease onset.

## 2. Methods

### 2.1. Participants

The participants selected for this study were also included in a previous work from our group on visual retinotopy [6]. We included 13 RP individuals (8 males and 5 females; mean age 38.31 ± 12.65 years; age range 20 - 66 years; 12 right-handed and 1 left-handed; self-reported symptomatic age of onset range 2 - 39 years, resulting in symptomatic duration range 6 - 42 years) and 22 control subjects (11 males and 11 females; mean age 38.45 ± 12.29 years; age range 23 - 66 years; 21 right-handed and 1 left-handed). Both groups were matched for age (*t*_(33)_ = ‐0.03, nonsignificant *p* > 0.050 (NS)), gender (*χ*^2^_(1)_ = 0.44, NS), and handedness (*χ*^2^_(1)_ = 0.15, NS) ratio. Patients were recruited at the Centro Hospitalar e Universitário de Coimbra. The control group participants were local volunteers. The study was conducted in accordance with the Declaration of Helsinki and was approved by the Ethics Committee of Faculty of Medicine of the University of Coimbra. Written informed consent was obtained from all participants. Exclusion criteria were intracranial abnormalities, movement during MRI acquisitions, fixation instability, visual alterations in control subjects, or visual alterations other than RP for patients (e.g., diabetic retinopathy or glaucoma).

### 2.2. Ophthalmological Assessment

**Figure 1:**
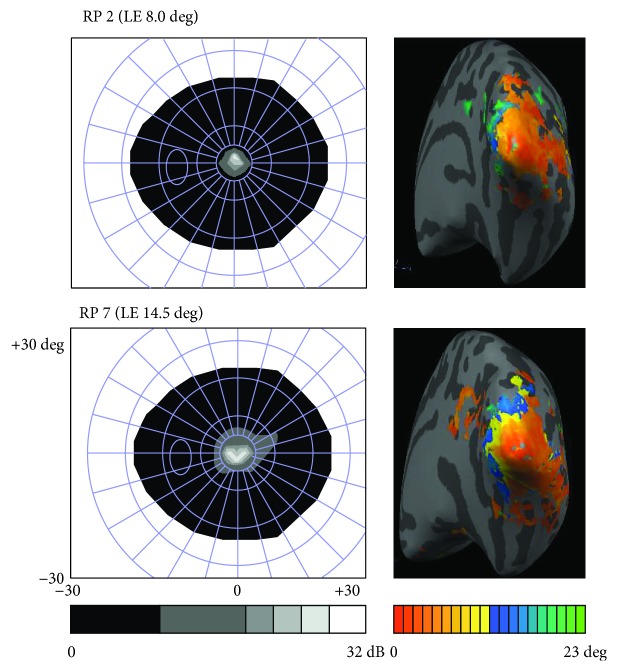
Representation of the left eye lesions (scotomata) measured with static perimetry on the left side of the figure (gray scale represents visual field sensitivity in dB), and right hemisphere retinotopic eccentricity maps on the right side of the figure (colored axis represents visual field extent in degrees (1 to 23 deg); Linear Correlation Maps, *r* > 0.25; inflated hemisphere mesh) in two patients. RP = Retinitis Pigmentosa and LE = left eye.

For each participant, we measured visual acuity with a decimal chart (converted to logarithm of Minimum Angle of Resolution (logMAR) scale), average cortical thickness, and retinal nerve fiber layer (RNFL) thickness with Frequency Domain Cirrus Ocular Coherence Tomography (OCT, software version 5.1.1.6, Carl Zeiss Meditec AG, USA), and static visual fields with a MonCv3 multifunction perimeter (Metrovision, France) (Figure 1). A detailed description of the methodology used was described in our previous study [6] (Table 1).

### 2.3. Brain Imaging Procedures

Scanning was performed on a 3 T scanner (Magneton TrioTim, Siemens AG, Germany) at the Portuguese Brain Imaging Network, using a 12-channel birdcage head coil. Two anatomical T1-weighted Magnetization-Prepared Rapid Acquisition with Gradient Echo (MPRAGE) sequences with 1 × 1 × 1 mm^3^ voxel size, Repetition Time (TR) 2.53 s, Echo Time (TE) 3.42 ms, Flip Angle (FA) 7°, Field Of View (FOV) 256 × 256 mm^2^, and 176 slices were acquired from each participant. Functional sequences consisted of a single shot Echo-Planar Imaging (EPI) acquired in the axial plane parallel to the Anterior Commissure (AC)-Posterior Commissure (PC) plane with 2 × 2 × 2 mm^3^ voxel size, TR 2 s, TE 39 ms, interslice time (TI) 76 ms, FA 90°, FOV 256 × 256 mm^2^, 26 slices, and 128 × 128 imaging matrix.

Stimuli were presented using MRI compatible goggles with refractive correction (VisualSystem, NordicNeurolab, Norway). One eye was covered with a cotton patch while the other received the visual input (the dominant eye, except if it was the eye with the worst visual acuity). The RP and control group were matched for the selected eye ratio (*χ*^2^_(1)_ = 0.85, NS) and for the dominance of the selected eye ratio (*χ*^2^_(1)_ = 0.01, NS; 1 patient with missing data). The maximum field of view was 23 × 30 deg (resolution of 600 × 800).

### 2.4. MRI Stimuli

Stimuli were designed using Matlab 2011b (the MathWorks Inc., USA) with Psychophysics Toolbox 3 extensions (http://psychtoolbox.org/). A central red-colored cross with 0.78 deg of diameter was used for fixation [6].

#### 2.4.1. Retinotopic Mapping Stimuli

Polar angle and eccentricity stimuli were employed to delineate the cortical visual areas (V1, V2, and V3) using the traveling-wave approach from the standard phase-encoded retinotopic mapping [27]. Polar angle maps were obtained using a black and white flickering checkerboard wedge with 45 deg rotating in an anticlockwise direction (initial angle of 22.50 deg with horizontal axis). Eccentricity maps were obtained using a black and white flickering checkerboard expanding ring (for additional details see [6]). Checkerboard size varied with cortical magnification factor from the center to the periphery [28, 29]. Stimuli flickering frequency was 8 Hz and contrast was ~100%. Each run comprised 2 baseline blocks (~0% contrast; 12 s) at the beginning and end of the run with 4 cycles of polar angle or eccentricity stimuli (48 s each; total duration of a run 216 s). Two runs of polar angle and 2 runs of eccentricity were acquired for each subject.

#### 2.4.2. Attentional Task Stimuli

**Figure 2:**
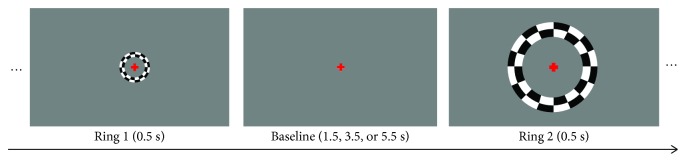
Representation of the task paradigm with the central (Ring_1_) and the paracentral (Ring_2_) flickering checkerboard rings and the interstimulus intervals. During the passive viewing condition, participants had to fixate the central red cross. During the visual memory task condition, participants pressed a button every time a repeated ring appeared (one-back task). Scale of the fixation dot has been changed to enhance visibility.

The set of stimuli of the main experimental task of this study consisted of a random sequence of 2 different sized checkerboard rings pseudorandomly presented during either passive viewing (fixation only) or a one-back visual memory task condition. During the task condition, participants were instructed to press a button every time a ring was the same size as the immediately preceding one. Ring_1_ was presented at a foveal location (diameter between 0.78 and 1.90 deg), and Ring_2_ at a parafoveal location of the visual field (diameter between 6.74 and 9.52 deg). Ring thickness varied with the cortical magnification factor from the center to the periphery [28, 29]. Rings appeared during 0.50 s in random intervals of 1.50, 3.50, or 5.50 s within each block. Four passive viewing blocks and 4 task blocks (~100% contrast; flickering frequency of 8 Hz; 10 rings; 36 s) were alternately presented, intercalated with 9 baseline blocks (~0% contrast; 12 s; each run began and ended with one baseline block). Auditory instructions were provided to the subject before each block, depending on the condition: “Rest” for passive viewing or “Answer” for the one-back task. The average response time and percentage of errors during the task were recorded. Participants who answered during passive viewing blocks were excluded.  Figure 2 shows a representation of the one-back task stimulus. We aimed at performance matching between patients and controls. We preferred this choice as compared to a possible 2-back task, because we believe that this would lead to quite large executive load.

### 2.5. MRI Data Processing

Brain imaging analysis was performed with BrainVoyager QX 2.6.1 (Brain Innovation B. V., Netherlands). The two anatomical images of each participant were averaged, reoriented to AC–PC plane, and transformed to Talairach (TAL) space. The image was segmented into cerebral spinal fluid, gray matter, and white matter to create inflated mesh representations of each hemisphere. A cut was manually drawn along the calcarine sulcus, and meshes were flattened for retinotopic maps projection [6, 30].

The preprocessing of functional sequences consisted of scan time correction, temporal high-pass filtering (2 cycles per run), spatial smoothing (FWHM 2 mm), and a correction for small interscan head movements. Participants were excluded if within-run movements exceeded 4 mm (-2 to 2 mm). Polar angle and eccentricity maps were obtained from the average of the two runs, created based on linear regression analysis, and projected onto the flattened surfaces of each subject (statistical maps with *r* > 0.25; Figure 1) [6]. Field Sign Maps were automatically created using polar angle and eccentricity Look-Up Table maps. Retinotopic areas V1 dorsal (V1_d_), V1 ventral (V1_v_), V2 dorsal (V2_d_), V2 ventral (V2_v_), V3 dorsal (V3_d_), and V3 ventral (V3_v_) were manually defined for each subject in each hemisphere on flattened meshes.

**Figure 3:**
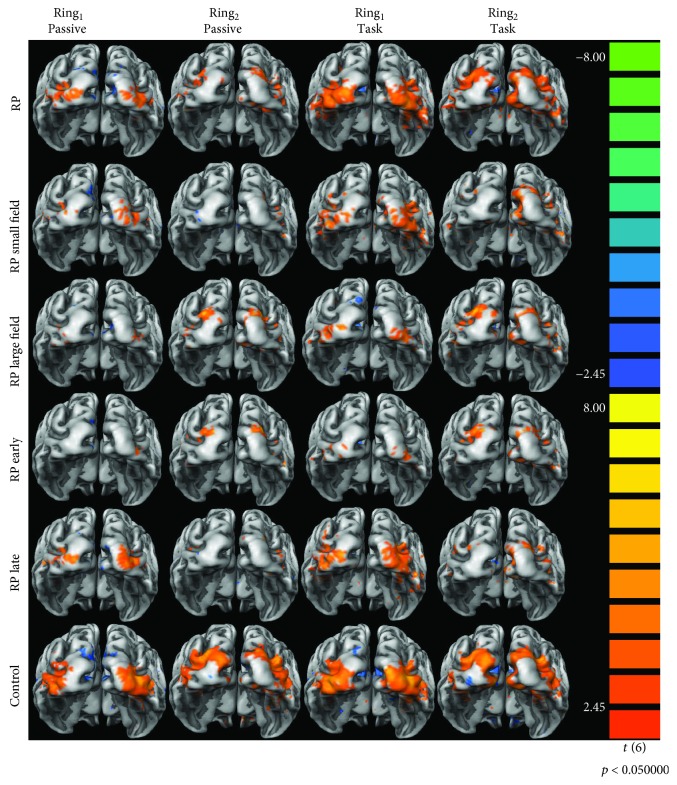
Representation of the visual cortical responses for all the analyzed general linear model predictors (*Ring_1_ Passive Viewing*, *Ring_2_ Passive Viewing*, *Ring_1_ Task*, and *Ring_2_ Task*) for the Retinitis Pigmentosa group (RP) and the control group. The visual cortical activation is also represented for the subgroups of patients RPsvf (RP small field) with less than 9.52 deg of visual field diameter and RPlvf (RP large field) with more than 9.52 deg of visual field diameter. Finally, the visual cortical responses are also displayed for the subgroups of patients RPeo (RP early) with disease onset ages lower than 14 years and RPlo (RP late) with onset ages higher or equal to 14 years. Images represented the posterior view of both hemispheres meshes averaged for all participants. The colored scale represents the *t*-test value for the contrast predictor versus baseline with *p* < 0.050.

Statistical analyses were performed on individual data in TAL space using a general linear model (GLM) (*z*-transformation, False Discovery Rate (FDR) *q* < 0.05, correction for temporal serial correlations AR(2)) within the retinotopically defined visual areas. Response predictors for the visual memory task were obtained, and beta values evoked by each stimulus conditions were retrieved: *Ring_1_ Passive Viewing*, *Ring_2_ Passive Viewing*, *Ring_1_ Task*, and *Ring_2_ Task*.  Figure 3 represents the visual cortical responses for all predictors during the visual memory task for RP patients and control participants.

**Figure 4:**
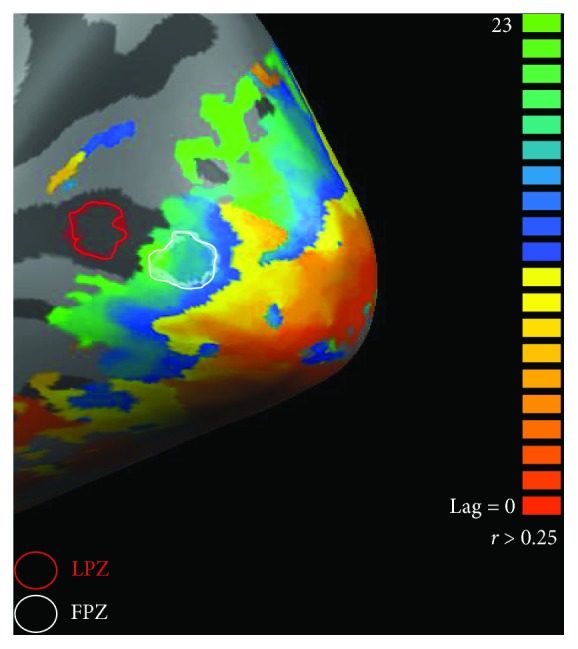
Representation of the cortical regions of interest—the function projection zone (FPZ) and lesion projection zone (LPZ)—on the right hemisphere of a control participant. FPZ represents the preserved visual field region and the LPZ represents the visual field scotomata in patients or the unstimulated visual field in controls. These cortical regions were manually defined along the calcarine sulcus (V1) considering the retinotopic eccentricity map.

A multistudy GLM (random fixed effects, *z*-transformation, FDR *q* < 0.05, AR(2)) was also run in two different regions of interest along the calcarine sulcus (V1). The functional projection zone (FPZ) represented the preserved visual field region, and the LPZ represented either the visual field scotomata in patients or the unstimulated visual field in controls. These cortical regions were manually defined considering the retinotopic eccentricity maps of each participant.  Figure 4 illustrates the location of the two regions of interest, the FPZ and the LPZ, in one control participant. Response predictors for the visual working memory task were obtained, and beta values evoked by each stimulus conditions were retrieved inside these regions of interest: *Ring_1_ Passive Viewing*, *Ring_2_ Passive Viewing*, *Ring_1_ Task*, and *Ring_2_ Task*.

Cortical thickness was calculated on the retinotopic areas using the standard procedure of BrainVoyager (see [30] for a complete description). To allow an accurate segmentation of white matter–gray matter and gray matter–cerebral spinal fluid boundary, TAL anatomical data were converted to high-resolution 0.5 × 0.5 × 0.5 mm^3^. The subcortical structures and the ventricles were filled as white matter. After computation, cortical thickness maps were superimposed on cortical meshes, and mean cortical thickness values of all visual areas were extracted using Matlab BVQXtools toolbox extensions (http://support.brainvoyager.com/available-tools/52-matlab-tools-bvxqtools.html).

### 2.6. Subgroup Analysis

To understand the influence of the level of peripheral degeneration and disease onset age on visual cortex response, the RP group was divided accordingly to these factors. The RP group was divided accordingly to the extent of visual field measured by the static perimetry test in two subgroups: RPsvf (small visual field) (*n* = 6, patients RP 1 to RP 6) with bilateral visual field diameter under 9.52 deg and RPlvf (large visual field) (*n* = 7, patients RP 7 to RP 13) with bilateral visual field diameter over 9.52 deg (see Table 1). In this way, the RPlvf patients were expected to see the complete visual working memory task stimuli (Ring_1_ and Ring_2_, because the maximum diameter of Ring_2_ was 9.52 deg), whereas most RPsvf patients would only partially see Ring_2_. Both subgroups and the control group were matched for age (*F*_(2, 32)_ = 1.00, NS), gender (*χ*^2^_(2)_ = 0.56, NS), handedness (*χ*^2^_(2)_ = 1.37, NS), selected eye (*χ*^2^_(2)_ = 1.44, NS), and selected eye dominance ratio (*χ*^2^_(2)_ = 1.65, NS; 1 patient with missing data). Additionally, the disease onset age (*F*_(1, 11)_ = 2.80, NS) and the disease symptomatic duration (*F*_(1, 11)_ = 0.00, NS) were not different between the subgroups RPsvf and RPlvf.

The RP group was also divided accordingly to the disease onset age into two subgroups: RPeo (early onset) (*n* = 6, patients RP 5, 7, 8, 9, 11, and 13) with an onset age lower than 14 years and RPlo (late onset) (*n* = 7, patients RP 1, 2, 3, 4, 6, 10, and 12) with an onset age greater than or equal to 14 years (see Table 1). Patients from subgroup RPeo with earlier disease onset age were expected to have more prominent alterations in visual cortex responses than RPlo patients since some visual plasticity is thought to remain until 14 to 16 years of age [8, 31]. Both subgroups and the control group were matched for age (*F*_(2, 32)_ = 0.43, NS), gender (*χ*^2^_(2)_ = 0.57, NS), handedness (*χ*^2^_(2)_ = 1.82, NS), selected eye (*χ*^2^_(2)_ = 2.96, NS), and selected eye dominance ratio (*χ*^2^_(2)_ = 0.01, NS; 1 patient with missing data). As expected, the disease onset age (*F*_(1, 11)_ = 19.29, *p* = 0.001) was different between the two subgroups of patients RPeo and RPlo, but the disease symptom duration (*F*_(1, 11)_ = 2.81, NS) was not different.  Table 2 summarizes the information of visual field extent and onset age for all subgroups.

### 2.7. Statistical Analysis

Statistical analysis was performed with IBM SPSS Statistics, Version 22 (IBM Corporation, USA). Normality and homogeneity of variance were tested using Shapiro-Wilk's test and Levene's test, respectively. For data in accordance with these assumptions, statistical parametric tests were performed. Otherwise, nonparametric methods were applied. Bonferroni correction was applied for multiple comparisons (*p* values presented as *correctedp*). The epsilon value was used for correction of nonspherical data (Huynh-Feldt for epsilon higher than 0.75 and Greenhouse-Geisser for epsilon lower than 0.75). The significance level was 0.05, and the statistical power was higher than 0.80 for all presented results.

## 3. Results

### 3.1. Visual Assessment

The first two parts of the results section (Visual Assessment and Behavioral Data) are focused on ophthalmological features—visual acuity, visual field extent, average retinal thickness, RNFL thickness-, and performance in visual memory task in patient and clinical groups and subgroups. The subsequent parts are focused on the main hypotheses of the article.

#### 3.1.1. All Groups (No Stratification according to Visual Field Extent or Age of Onset)

Visual acuity (left eye (LE) *U* = 275.00, *p* = 2.642 × 10^−7^ and right eye (RE) *U* = 286.00, *p* = 1.355 × 10^−9^) and average retinal thickness (LE *U* = 275.00, *p* = 1.883 × 10^−7^ and RE *U* = 283.00, *p* = 9.483 × 10^−9^) were reduced in both eyes for patients as compared to control participants. Visual field deficit volume (LE *U* = 3.00, *p* = 9.483 × 10^−9^ and RE *U* = 2.00, *p* = 5.419 × 10^−9^) was higher in both eyes for patients as compared to the control group. No differences were found for RNFL thickness in both eyes between the two groups (LE *U* = 125.00, NS and RE *U* = 120.00, NS). Moreover, no statistically significant differences were found between the left and right eyes within groups for visual acuity (RP *Z* = 1.33, NS and control *Z* = 1.41, NS), average retinal thickness (RP *Z* = −0.84, NS and control *Z* = −0.88, NS), RNFL thickness (RP *Z* = 0.00, NS and control *Z* = −0.07, NS), visual field deficit volume (RP *Z* = −1.24, NS and control *Z* = −0.28, NS), and visual field extent (RP *Z* = −1.18, NS; controls have a constant visual field extent equal to 48 deg corresponding to the maximum diameter covered by our static perimetry) [6].

#### 3.1.2. Visual Field (Large vs. Small Visual Field) Subgroup Analysis

Visual acuity (LE *χ*^2^_(2)_ = 22.62, *p* = 1.200 × 10^−5^ and RE *χ*^2^_(2)_ = 25.64, *p* = 3.000 × 10^−6^) and average retinal thickness (LE *χ*^2^_(2)_ = 20.91, *p* = 2.900 × 10^−5^ and RE *χ*^2^_(2)_ = 23.01, *p* = 1.000 × 10^−5^) were reduced in both eyes for the two RP subgroups as compared to control participants. No differences were found for RNFL thickness in both eyes between the three groups (LE *χ*^2^_(2)_ = 3.00, NS and RE *χ*^2^_(2)_ = 0.65, NS). Visual field deficit volume (LE *χ*^2^_(2)_ = 23.00, *p* = 1.000 × 10^−5^ and RE *χ*^2^_(2)_ = 23.29, *p* = 9.000 × 10^−6^) was higher in both eyes for both patients' subgroups as compared to the control group. According to the subgroup division, visual field extent was different between the two subgroups (LE *χ*^2^_(1)_ = 9.10, *p* = 0.003 and RE *χ*^2^_(1)_ = 9.02, *p* = 0.003), with RPsvf (small visual field) patients presenting smaller visual fields than RPlvf (large visual field/more preserved). Moreover, no statistically significant differences were found between the left and right eyes within groups for visual acuity (RPsvf *Z* = 0.73, NS, RPlvf *Z* = 1.26, NS, and control *Z* = 1.41, NS), average retinal thickness (RPsvf *Z* = 1.15, NS, RPlvf *Z* = 0.42, NS, and control *Z* = 0.88, NS), RNFL thickness (RPsvf *Z* = 1.58, NS, RPlvf *Z* = −1.76, NS, and control *Z* = −0.07, NS), visual field deficit volume (RPsvf *Z* = −0.36, NS, RPlvf *Z* = −1.35, NS, and control *Z* = −0.28, NS), and visual field extent (RPsvf *Z* = −0.73, NS and RPlvf *Z* = −0.95, NS; controls have a constant visual field extent equal to 48 deg corresponding to the maximum diameter covered by static perimetry).

#### 3.1.3. Age of Onset Subgroup Analysis

Visual acuity (LE *χ*^2^_(2)_ = 22.55, *p* = 1.300 × 10^−5^ and RE *χ*^2^_(2)_ = 25.42, *p* = 3.000 × 10^−6^) and average retinal thickness (LE *χ*^2^_(2)_ = 20.77, *p* = 3.100 × 10^−5^ and RE *χ*^2^_(2)_ = 22.89, *p* = 1.100 × 10^−5^) were reduced in both eyes for the two RP subgroups (RPeo, early onset; RPlo, late onset) as compared to control participants. No differences were found for RNFL thickness in both eyes between the three groups (LE *χ*^2^_(2)_ = 0.38, NS and RE *χ*^2^_(2)_ = 1.48, NS). Visual field deficit volume (LE *χ*^2^_(2)_ = 22.88, *p* = 1.100 × 10^−5^ and RE *χ*^2^_(2)_ = 23.19, *p* = 9.000 × 10^−6^) was higher in both eyes for both patients' subgroups as compared to the control group. The visual field extent was not different between the two subgroups of patients (LE *χ*^2^_(1)_ = 2.50, NS and RE *χ*^2^_(1)_ = 2.95, NS). Moreover, no statistically significant differences were found between the left and right eyes within groups for visual acuity (RPeo *Z* = 1.08, NS, RPlo *Z* = 0.13, NS, and control *Z* = 1.41, NS), average retinal thickness (RPeo *Z* = −0.21, NS, RPlo *Z* = −1.18, NS, and control *Z* = −0.88, NS), RNFL thickness (RPeo *Z* = −1.21, NS, RPlo *Z* = −1.16, NS, and control *Z* = −0.07, NS), visual field deficit volume (RPeo *Z* = −0.40, NS, RPlo *Z* = −1.36, NS, and control *Z* = −0.28, NS), and visual field extent (RPeo *Z* = −0.52, NS and RPlo *Z* = −1.29, NS; controls have a constant visual field extent equal to 48 deg corresponding to the maximum diameter covered by static perimetry).

In sum, ophthalmological tests showed decreased patients' visual acuity, visual field extent, and average retinal thickness, while RNFL thickness was preserved. Importantly, subgroups defined by visual field extent and age of onset only differ in the visual field extent or in the age of onset of the disease, respectively.  Table 2 shows a summary of participants' ophthalmologic characterization, presenting the visual parameter values for each group and subgroups.

### 3.2. Visual Memory Task: Behavioral Data

The mean response time (*U* = 110.00, NS) and response error (*U* = 99.00, NS) were not different between the RP and control groups during the performance of the one-back visual memory task, and both groups were actually near ceiling levels. Concerning the visual field subgroups, we did not find differences among the RPsvf and RPlvf subgroups and the control group in response time (*F*_(2, 32)_ = 0.03, NS) and error (*F*_(2, 32)_ = 1.00, NS). For the onset age subgroups, the analysis did not show differences among the RPeo and RPlo subgroups and the control group in response time (*F*_(2, 32)_ = 0.02, NS) and error (*F*_(2, 32)_ = 0.23, NS). Groups were therefore behaviorally matched.

### 3.3. Visual Memory Task: Responses in Functional (FPZ) and Lesion Projection Zone (LPZ)

Here, we tested the hypothesis that compensatory allocation of visual attention mechanisms do occur in patients.

#### 3.3.1. All Group

The beta values of the task predictors (Ring_1_ Passive Viewing, Ring_2_ Passive Viewing, Ring_1_ Task, and Ring_2_ Task) were analyzed between groups for each condition (*Condition* Passive Viewing and Task), each ring (*Ring* Ring_1_ and Ring_2_), each cortical zone (*Zone* FPZ and LPZ in V1, see Figure 4), and each hemisphere (*Hemisphere* Left and Right) with repeated measures ANOVA. We found a significant effect of the predictor beta values between groups (*F_(1,33)_* = 4.47, *p* = 0.042). Importantly, within-subject effects for *Condition* (*F_(1,33)_* = 97.18, *p* = 2.313 × 10^−11^) and *Condition×Group* (*F_(1,33)_* = 9.66, *p* = 0.004) were present. To analyze the effect of the interaction *Condition×Group*, we used ANOVA between groups for each condition (Passive Viewing and Task). Results showed that the cortical responses during the task were higher for RP patients when compared to the control group (*p* = 0.002), whereas no differences were found for passive viewing condition between groups, thereby corroborating the main hypothesis.

#### 3.3.2. Analyses by Subgroups Defined by Extent of Visual Field Loss

The statistical analysis described above was conducted to study differences among the two subgroups of patients according to the extent of visual field loss and the control group. Importantly, we found the effects for *Condition* (*F_(1,32)_* = 135.11, *p* = 5.054 × 10^−13^), *Condition×Group* (*F_(2,32)_* = 13.86, *p* = 4.600 × 10^−5^), *Zone×Ring* (*F_(1,32)_* = 11.52, *p* = 0.002), and *Zone×Ring×Group* (*F_(2,32)_* = 6.86, *p* = 0.003). ANOVA for each condition (Passive Viewing and Task) was applied to analyze the interaction effect of *Condition×Group*. Results showed that the cortical responses for task were higher for RPsvf (small visual field) patients when compared to the RPlvf (large visual field) patients (*correctedp* = 0.013) and the control group (*correctedp* = 8.500 × 10^−5^), whereas no differences were found for passive viewing condition among the three groups. In this way, patients with smaller intact visual fields presented higher cortical response during the task than patients with larger visual field extent and controls. To study the effect of the interaction *Zone×Ring×Group*, we used ANOVA among groups for values of the ring (Ring_1_ and Ring_2_) in each cortical zone (FPZ and LPZ). We found increased cortical responses for RPsvf patients for Ring_2_ in the FPZ when compared to controls (*correctedp* = 0.014). In this way, patients with smaller visual fields presented higher cortical response (2.37 ± 2.21) than controls (−0.72 ± 2.21) in the FPZ during the stimulation of paracentral visual field, while patients with larger visual field extent (0.06 ± 2.21) had similar activation to controls. These observations suggest that larger damage (present in small visual field patients) lead to larger allocation of visual attention mechanisms.

#### 3.3.3. Analyses Based on Age of Onset Defined Subgroups

The statistical analysis described above was conducted to study differences among the two subgroups of patients according to the age of disease onset and the control group. Effects for *Condition* (*F_(1,32)_* = 90.76, *p* = 7.325 × 10^−11^) and *Condition×Group* (*F_(2,32)_* = 5.35, *p* = 0.010) were present. ANOVA for each condition (Passive Viewing and Task) was applied to analyze the interaction effect of *Condition×Group*. Results showed that the cortical responses for the task were surprisingly higher for RPlo (later onset) patients when compared to the control group (*correctedp* = 0.006), whereas no differences were found for passive viewing condition among the three groups. In sum, patients with later disease onset presented surprisingly higher cortical response during task than controls, suggesting that lower activation may represent more “efficient” brain activity patterns in early onset patients (with more prolonged disease evolution and more time to recruit mechanisms where “efficiency” dominates).

### 3.4. Visual Memory Task: Responses in Retinotopic Regions (V1, V2, and V3)

In this section, we investigate how different retinotopic areas contribute to the observed patterns of activity (overall and according to the subgroups defined above).

#### 3.4.1. All Group

The beta values of the task predictors (*Ring_1_ Passive Viewing*, *Ring_2_ Passive Viewing*, *Ring_1_ Task*, and *Ring_2_ Task*) were analyzed between groups for each condition (*Condition* Passive Viewing and Task), each ring (*Ring* Ring_1_ and Ring_2_), each visual area (*Area* V1, V2, and V3), each cortical visual region (*Region* Ventral and Dorsal), and each hemisphere (*Hemisphere* Left and Right). We found the main effects of *Area* (*F_(1.74,57.42)_* = 50.45, *p* = 1.700 × 10^−12^), *Condition* (*F_(1,33)_* = 44.27, *p* = 1.427 × 10^−7^), and *Ring* (*F_(1,33)_* = 53.81, *p* = 2.019 × 10^−8^). We also found the effects of *Area×Condition* (*F_(1.76,58.20)_* = 25.22, *p* = 4.532 × 10^−8^), *Area×Region×Ring* (*F_(1.72,56.92)_* = 9.12, *p* = 0.001), and *Area×Region×Ring×Group* (*F_(1.72,56.92)_* = 5.98, *p* = 0.006). We can summarize these interactions by the change in visual cortical responses from V1 to V3 (*Area*) between passive viewing and the one-back task condition (*Condition*) and between Ring_1_ (central visual field) and Ring_2_ (paracentral visual field) (*Ring*) for both groups.

We then hypothesized that age of onset and extent of visual field loss could influence the effects in patients.

#### 3.4.2. Visual Field Subgroup

The statistical analysis described above was conducted to study differences between the two subgroups of patients accordingly to the extent of visual field loss and the control group. We found the effects for *Area* (*F_(1.82,58.10)_* = 33.96, *p* = 5.461 × 10^−10^), *Condition* (*F_(1,32)_* = 58.91, *p* = 9.513 × 10^−9^), *Ring* (*F_(1,32)_* = 48.37, *p* = 7.057 × 10^−8^), *Condition×Group (F_(2,32)_* = 8.53, *p* = 0.001), *Area×Condition* (*F_(1.80,57.65)_* = 20.06, *p* = 5.902 × 10^−7^), *Area×Hemisphere×Group* (*F_(3.95,63.20)_* = 3.71, *p* = 0.009), and *Area×Region×Ring* (*F_(1.78,57.00)_* = 11.04, *p* = 1.590 × 10^−3^). An ANOVA for each condition (Passive Viewing and Task) was applied to analyze the critical interaction effect of *Condition×Group*. Results showed that the cortical responses for task were higher for RPsvf (small visual field) patients when compared to the control group (*correctedp* = 0.034), whereas no differences were found for the passive viewing condition among groups. In this way, patients with smaller visual fields presented higher cortical response during task than controls, while patients with larger visual field extent showed responses that were similar to the control participants. To study the effect of the interaction *Area×Hemisphere×Group*, we used ANOVA between groups for each visual area (V1, V2, and V3) in each hemisphere (Left and Right). We found increased cortical responses for RPsvf patients in the right V1 when compared to controls (*correctedp* = 0.044), but the remaining visual areas presented similar cortical activation. In this way, patients with smaller visual fields presented higher cortical response in the right primary visual cortex (3.17 ± 1.93) when compared to controls (0.87 ± 1.93), while patients with larger visual field extent (1.07 ± 1.93) showed similar activation to controls.

#### 3.4.3. Subgroup Defined by Distinct Age of Onset

The statistical analysis described above was conducted to study differences among the two subgroups of patients according to the age of onset and the control group. We found the effects for *Area* (*F_(1.89,60.64)_* = 42.83, *p* = 5.413 × 10^−12^), *Condition* (*F_(1,32)_* = 40.44, *p* = 3.853 × 10^−7^), *Ring* (*F_(1,32)_* = 51.74, *p* = 3.615 × 10^−8^), *Area×Group* (*F_(3.79,60.64)_* = 4.46, *p* = 0.004), *Area×Condition* (*F_(1.82,58.365)_* = 20.28, *p* = 4.572 × 10^−7^), *Area×Hemisphere×Group* (*F_(3.96,63.40)_* = 3.99, *p* = 0.006), *Region×Ring×Group* (*F_(2,32)_* = 5.66, *p* = 0.008), and *Area×Region×Ring* (*F_(1.79,57.38)_* = 11.51, *p* = 1.100 × 10^−4^).

In sum, this analysis focused on retinotopic areas that essentially mimics the findings observed for the LPZ and FPZ zones, corroborating the main hypothesis of preferential attentional allocation in patients, and distinct effects of the visual field lesion extent and age of onset.

### 3.5. Cortical Thickness of Visual Areas (V1, V2, and V3)

Finally, we investigated whether functional changes were associated with structural alterations.

Visual cortical thickness differences were evaluated using repeated measures ANOVA with three within-subject factors (*Area* V1-V3, *Region* Ventral vs Dorsal, and *Hemisphere* Left Vs Right), one between-subject factor *Group* (RP vs. Control), with the average brain cortical thickness as a covariate to account for variability across participants. Cortical thickness of the individually defined visual areas was not different between the two groups (*F_(1,32)_* = 0.047, NS), and no within-subject effects or interactions were found. This was also true for subgroup analyses.

## 4. Discussion

We investigated whether visual cortical responses in a disorder of peripheral vision are related to recruitment of attentional mechanisms. To test this hypothesis, we used a visual one-back task and passive viewing conditions with a visual stimulus covering the central (Ring_1_) and paracentral (Ring_2_) visual field in a group of RP patients (*n* = 13) with peripheral retinal loss and matched healthy controls (*n* = 22). Cortical responses were studied in visual retinotopic areas (V1, V2, and V3) and in two different regions of interest in V1: the FPZ representing the preserved visual field and the LPZ representing the visual field scotomata. To understand the influence of the level of peripheral degeneration and the disease age of onset, the analysis was further conducted for two distinct RP subgroups: subcategories defined by the extent of visual field loss (RPsvf, remaining small field, and RPlvf, remaining large field patients with bilateral visual field diameters under or over 9.50 deg, respectively), and subgroups defined by distinct ages of disease onset (RPeo, early onset, and RPlo, late onset—patients with age of onset of the disease lower or equal/greater than 14 years, respectively).

Our results demonstrated that RP patients have overall preserved visual cortical responses under central and paracentral visual field stimulation. Visual cortical responses (V1, V2, and V3) to the visual memory task stimuli were also overall preserved. A critical interaction with task condition was however found: RP patients presented higher overall cortical responses during the task condition than control participants in FPZ and LPZ regions. This was further highlighted when extent of visual loss was taken into account.

Concerning the role of the extent of visual loss, RPsvf patients, with smaller visual fields, presented higher overall visual cortical responses during the task condition than control participants, while responses for RPlvf patients, with larger visual field extent, were similar to control participants. Additionally, RPsvf patients had significantly higher cortical activation in the right V1 when compared to controls, while responses for RPlvf patients were similar to healthy participants. In line with these results, RPsvf patients presented higher overall cortical responses during the task condition than control participants and RPlvf patients in LPZ and FPZ regions, while responses for RPlvf patients were similar to control participants. Additionally, the cortical activation in the FPZ was higher for RPsvf patients during the stimulation of the paracentral visual field (Ring_2_) when compared to the healthy subjects. Because visual stimuli were the same in both task and passive viewing conditions, these responses for RPsvf patients seem to be related to attentional demands during the one-back task. Masuda et al. [13] found a similar increase in striate cortical responses of three RP participants related to changes in task demands and suggested that unmasking of feedback signals from the extrastriate cortex occurs when retinal signals are absent. Such unmasking might come from activation of previously silent synapses [32]. These feedback signals might be associated with attention, visual imagery, and task-related visual processing [26]. In our work, enhanced attentional top-down modulation may compensate for the lack of retinal input from the peripheral visual field in RPsvf patients with greater visual field loss [9, 24, 33]. A recent study presented evidence for increased functional connectivity between afferent early visual areas and cortical regions involved in visual processing (middle occipital gyrus and superior temporal gyrus/sulcus) in RP patients, suggesting a possible compensatory mechanism for peripheral visual loss. These authors also found enhanced functional connectivity between the deafferented visual cortex and higher-order regions (inferior parietal lobe/sulcus and middle frontal gyrus) involved in top-down control, attentional processes, and multisensory integration [34].

However, in the three patients reported in Masuda et al.'s work, the increased responses during task were found in the V1 LPZ [13]. Here, we report an overall increase in cortical responses under task demands while analyzing V1, V2, V3, and V1 FPZ and LPZ for patients with more severe visual field degeneration. In this way, feedback signals might influence both striate and extrastriate visual cortex when there is a severe lack of peripheral retinal input, not being restricted to the LPZ. A recent work from our group provided evidence of functional remapping in V1 in the same group of patients studied here. This functional reorganization was also more prominent in RP patient with larger visual field damage [6].

Previous works with macular degeneration also showed increased cortical responses for V1 LPZ while patients performed a one-back task with peripheral stimulation [15–19, 24], contrary to passive viewing stimulation [19, 21–25]. Some of the authors showed that these V1 responses were higher for more severe central retinal loss without foveal sparing [15, 18], in accordance with our work. Recently, Plank et al. [26] reported that patients with central scotomata presented enhanced cortical activation in areas beyond the retinotopic cortex for complex images with naturalist scenes, supporting an increased top-down modulation of the deprived visual cortex. This result was further supported by the work of Sabbah et al. [34] showing increased functional connectivity between the LPZ and high-level regions in central retinal disease patients.

Several studies with glaucoma patients found reduced amplitude of cortical responses in V1 during passive viewing stimulation associated with structural damage of the optic disk, the RNFL thickness, and/or the visual field scotomata [35–39]. A recent study demonstrated reduced cortical activity within the LPZ in V1 and V2 in glaucoma patients under passive viewing [40]. Here, we did not find decreased activity during passive viewing in the cortical regions studied, which might indicate that RP patients have preserved visual cortical responses even for severe visual field damage, possibly due to a similar compensation mechanism by increased attentional modulation.

The cortical responses in visual areas were not globally significantly different among the RPeo and RPlo patients' subgroups with different onset ages and the control participants. Thus, overall visual cortical responses do not seem to be influenced by disease onset age, in contrast to the extent of visual loss. However, RPlo patients with later disease onset presented higher overall cortical responses during the task condition than control participants in FPZ and LPZ regions, while responses for RPeo patients with earlier forms of the disease were similar to control participants. This result was unexpected considering our initial hypothesis that earlier onset ages would lead to larger brain alterations. Two sorts of mechanisms might be operating: the first requiring long-term circuit modifications and, more present in early-onset patients, the second entailing stronger frontoparietal recruitment which tends to manifest more in patients with more recent changes in visual experience. There is indeed evidence showing that higher top-down modulation may indeed be stronger in participants with more recent changes in visual experience [41, 42]. Moreover, it may reflect the fact that longer disease durations may lead to efficient compensatory mechanisms and decrease of frontoparietal activation. A second study from Rosa et al. [41, 42] shows that frontoparietal activation decreases over time as patients' vision becomes more adapted. Less fMRI activation might actually indicate “more efficient” compensation [41, 42]. Studies in macular degeneration patients did not report the effects of the age of onset on visual cortical responses [15, 22], while others showed that juvenile-macular degeneration patients with earlier disease forms have stronger cortical activation than age-related macular degeneration patients with later onset age [19]. Future studies should address the discrimination between age-dependency of neuroplasticity and disease-duration effects, which are separable. Disease onset age is often difficult to determine, which can make this an imprecise measure of RP severity [11, 43].

Given the evidence for cortical reorganization in our prior study [6], remodeling at the retinal level is unlikely. This issue can be further clarified in the future by explicitly computing population receptive fields or alternatively running experiments with artificial scotomata.

Our results did not find evidence for visual cortical structural alterations in this cohort of RP patients, showing that the visual loss level was not sufficient to produce significant cortical atrophy in the visual areas studied (V1 to V3). To our knowledge, few structural MRI studies have been conducted with low vision RP patients [3, 44]. In our study, the RP patients did not present RNFL thickness atrophy which is in line with the preservation of visual cortical thickness. Nonetheless, in more advanced stages of RP disease with larger photoreceptor loss and retinal ganglion cell degeneration, disuse-driven mechanisms may lead to the visual cortical atrophy pattern that is often seen in macular degeneration, glaucoma, and also late-blindness.

## 5. Conclusion

We found that cortical visual areas (V1, V2, and V3) responses under attentional demands were increased in patients with larger degeneration of visual field. Moreover, activation during the task condition was increased for patients in both cortical regions corresponding to the preserved (FPZ) and the damaged visual field (LPZ), specifically for patients with severe visual field loss. These findings were identified in the presence of preserved visual cortical structure. The age of onset of the disease did not seem to be associated with visual cortical alterations. We conclude that RP patients may have relatively preserved visual cortical responses due to feedback attentional modulation in the absence of cortical atrophy, despite their retinal degeneration. The unmasking of corticocortical feedback signals from higher level visual regions involved in attentional processes might explain the increased cortical responses [1]. Such unmasking might lead to activation of previously silent synapses.

These results might be considered in the context of strategies for treating retinal diseases [21, 45, 46]. This is quite relevant given previous evidence that attentional cueing improves vision restoration therapy in patients with visual field loss [47, 48]. The role of higher-level neuronal networks [49] and their functional connectivity [50] cannot be underestimated in this context. This suggests that visual responses can be dynamically adapted as a function of flexible mechanisms requiring the interaction between high-level regions that implement attentional control.

## Figures and Tables

**Table 1:** Summary of the participants' characterization and ophthalmological test results for the Retinitis Pigmentosa group (adapted from [6]).

Patient	Age (years)	Gender	Eye dominance	Onset age (years)	Disease duration (years)	Visual acuity (logMAR)		Retinal thickness (*μ*m)		RNFL thickness (*μ*m)		Visual field deficit volume (dB·deg^2^)		Visual field extent (~diameter in deg)	
						LE	RE	LE	RE	LE	RE	LE	RE	LE	RE
RP 1	66	F	a	27	39	0.30	0.30	254.00	248.00	95.00	108.00	1349.00	1349.00	6.50	6.50
RP 2	42	M	LE	18	24	0.22	0.30	194.00	208.00	100.00	116.00	1718.00	1757.00	8.00	4.50
RP 3	50	M	RE	16	34	0.30	0.30	201.00	203.00	70.00	62.00	1168.00	1156.00	8.00	8.00
RP 4	23	M	RE	16	7	0.30	0.18	254.00	266.00	130.00	143.00	1240.00	1232.00	8.00	8.50
RP 5	35	M	LE	6	29	0.05	0.30	228.00	243.00	89.00	91.00	1700.00	1700.00	9.50	10.50
RP 6	45	F	LE	39	6	0.10	0.18	192.00	184.00	76.00	81.00	1204.00	1181.00	10.50	8.50
RP 7	20	M	LE	7	13	0.22	0.22	225.00	228.00	106.00	99.00	1639.00	1666.00	14.50	13.00
RP 8	35	F	RE	3	32	0.52	0.22	281.00	265.00	101.00	101.00	1650.00	1609.00	19.00	15.50
RP 9	50	M	LE	8	42	0.18	0.40	205.00	207.00	79.00	73.00	1414.00	1402.00	20.50	18.50
RP 10	38	F	RE	32	6	0.00	0.40	249.00	258.00	128.00	128.00	1520.00	1423.00	21.50	21.00
RP 11	38	F	RE	6	32	0.10	.55	216.00	221.00	133.00	109.00	1118.00	1030.00	23.00	29.00
RP 12	25	M	RE	14	11	0.40	0.30	242.00	245.00	101.00	95.00	802.00	539.00	43.00	43.00
RP 13	31	M	LE	2	29	0.10	0.40	273.00	268.00	91.00	92.00	72.00	126.00	47.50	47.00

RP = Retinitis Pigmentosa; F = female; M = male; LE = left eye; RE = right eye; RNFL = retinal nerve fiber layer; logMAR = logarithm of Minimum Angle of Resolution; ^a^missing information.

**Table 2:** Ophthalmological characterization of the participants from the patients' and controls' groups and for the patients from visual field extent subgroups (RPsvf and RPlvf) and the disease onset age subgroups (RPeo and RPlo). The visual field and onset age subgroups only differ in the visual field extent and in the age of onset of the disease, respectively. Results showed a severe decrease of patients' visual acuity, visual field extent, and average retinal thickness when compared to the control group (RNFL thickness was unchanged).

Visual parameters	Eye	RP group (*n* = 13)	Control group (*n* = 22)	Visual field extent		Disease age of onset	
				RPsvf subgroup (*n* = 6) < 9.50 deg	RPlvf subgroup (*n* = 7) > 9.50 deg	RPeo subgroup (*n* = 6) < 14 years	RPlo subgroup (*n* = 7) ≥ 14 years
Visual acuity (logMAR)	LE	0.22 (0.20)	0.00 (0.11)	0.26 (0.22)	0.18 (0.30)	0.14 (0.21)	0.30 (0.20)
	RE	0.30 (0.18)	0.00 (0.11)	0.30 (0.12)	0.40 (0.18)	0.35 (0.21)	0.30 (0.12)
Retinal thickness (*μ*m)	LE	228.00 (51.00)	288.50 (21.50)	214.50 (60.50)	242.60 (57.00)	226.50 (61.75)	242.00 (60.00)
	RE	243.00 (54.00)	285.00 (20.75)	225.50 (54.25)	245.00 (44.00)	235.50 (48.25)	245.00 (55.00)
RNFL thickness (*μ*m)	LE	100.00 (33.00)	94.50 (10.50)	92.00 (33.00)	101.00 (37.00)	96.00 (26.25)	100.00 (52.00)
	RE	99.00 (26.50)	95.00 (17.25)	99.50 (46.50)	99.00 (17.00)	95.50 (16.50)	108.00 (47.00)
Visual field deficit volume (dB·deg^2^)	LE	1349.00 (501.50)	30.00 (24.25)	1294.50 (509.50)	1414.00 (837.00)	1526.50 (806.00)	1240.00 (353.00)
	RE	1349.00 (544.50)	27.50 (44.00)	1290.50 (539.50)	1402.00 (1070.00)	1505.50 (870.50)	1233.00 (267.00)
Visual field extent (~diameter; deg)	LE	14.50 (14.25)	48^a^	8.00 (2.13)	21.50 (24.00)	19.75 (15.88)	8.00 (13.50)
	RE	13.00 (16.75)	48^a^	8.25 (3.00)	21.00 (27.50)	17.00 (21.13)	8.50 (14.50)
Onset age onset (years)	—	14.92 ± 11.58	—	20.33 ± 11.32	10.29 ± 10.34	5.33 ± 2.34	23.14 ± 9.63
Disease duration (years)	—	23.38 ± 13.07	—	23.17 ± 13.85	23.57 ± 13.48	29.50 ± 9.40	18.14 ± 14.09

Data are median (interquartile range) and mean ± standard deviation; RP = Retinitis Pigmentosa; RPsvf = small visual field; RPlvf = large visual field; RPeo = early onset; RPlo = late onset; LE = left eye; RE = right eye; RNFL = retinal nerve fiber layer; logMAR = logarithm of Minimum Angle of Resolution. ^a^Visual field extent for the control group is the maximum diameter tested during the static perimetry (48 deg).

## Data Availability

The data used to support the findings of this study are available from the corresponding author upon request.

## Abbreviations

RP: Retinitis Pigmentosa
MRI: Magnetic resonance imaging
fMRI: Functional magnetic resonance imaging
RNFL: Retinal nerve Fiber layer
LPZ: Lesion projection zone
FPZ: Function projection zone
deg: Degrees.
